# Analytical Chemistry of Impurities in Amino Acids Used as Nutrients: Recommendations for Regulatory Risk Management

**DOI:** 10.3390/nu14142838

**Published:** 2022-07-11

**Authors:** Sachise Karakawa, Miro Smriga, Naoko Arashida, Akira Nakayama, Hiroshi Miyano

**Affiliations:** 1 Research Institute for Bioscience Products & Fine Chemicals, Ajinomoto Co., Inc., Kawasaki 210-8681, Japan; naoko.arashida.2bq@asv.ajinomoto.com (N.A.); akira.nakayama.vy4@asv.ajinomoto.com (A.N.); miyano.hiroshi.cv3@asfrontiers.com (H.M.); 2 Quality Assurance Department, Ajinomoto Co., Inc., Tokyo 104-8315, Japan; miro.smriga.pb6@asv.ajinomoto.com

**Keywords:** amino acids, purity, analytical chemistry, international regulations, risk management

## Abstract

Proteinogenic amino acids are natural nutrients ingested daily from standard foods. Commercially manufactured amino acids are added to a wide range of nutritional products, including dietary supplements and regular foods. Currently, the regulatory risk management of amino acids is conducted by means of setting daily maximum limits of intake. However, there have been no reported adverse effects of amino acid overdosing, while impurities in low-quality amino acids have been identified as causative agents in several health hazard events. This paper reviews the analytical chemistry of impurities in amino acids and highlights major variations in the purity of commercial products. Furthermore, it examines the international standards and global regulatory risk assessment of amino acids utilized in dietary supplements and foods, recommending (1) further research on analytical methods that can comprehensively separate impurities in amino acids, and (2) re-focusing on the regulatory risk management of amino acids to the analytical chemistry of impurities.

## 1. Introduction

Amino acids are organic compounds containing amino (-NH2) and carboxyl (-COOH) functional groups combined with a side chain specific to each amino acid. Amino acids are chiral and can exist as L- or D-enantiomers, except for glycine, which has the simplest possible side chain. Hundreds of amino acids are known, although only 20 L-amino acids (proteinogenic amino acids) make up human proteins. This article addresses these proteinogenic L-amino acids (hereinafter “amino acids”).

Amino acids constitute human proteins, and they also have important physiological functions as free (non-protein bound) components of the diet [1]. For both of these reasons, many people choose to enhance their dietary intake of proteinogenic amino acids by means of dietary supplements (also called food supplements) or amino acid-fortified foods [2]. The effectiveness of such nutritional interventions depends on a plethora of factors, such as age, dose, background nutrition and physical activity, and is beyond the scope of this article.

Despite their broad range of uses in human nutrition, the quality and purity of commercially produced amino acids remain largely unregulated. Similar to other ingredients that are utilized without purity standards, these regulatory oversights may represent a health risk, as illustrated by the deadly eosinophilia-myalgia syndrome associated with an adulterated batch of the essential amino acid tryptophan in the early 1990s, e.g., [3,4,5] and a recent disease outbreak in Europe, the cause of which was linked to impure ingredients [6]. Although the causality of the most tragic cases of eosinophilia-myalgia syndrome in the 1990s remains obscure, its trigger was not the amino acid itself, but an impurity(s) contained in the adulterated batch [3]. There also remains an intriguing possibility that a health hazard may result from a specific impurity interacting with a specific amino acid, although there are insufficient experimental data to argue for such a case, e.g., [6].

Utilizing the case of amino-acid-containing products, we argue that controlling the purity of nutrients in dietary supplements and foods is the key enforceable factor in preventing adverse effects [6,7,8]. To do so, we briefly review the current regulatory status of proteinogenic amino acids, and the analytical methods applied for the evaluation of their purity. We also introduce a simple concept for the safe nutritional use of amino acids. For this review, we excluded essential amino acids that are added to infant products due to their well-defined regulatory status and different risk/benefit evaluation when compared to those applied to nutrients in products intended for older children and adults, e.g., [9].

## 2. Current Regulatory Status of Amino Acids Used as Nutrients

In Europe, amino acids are not specifically included in either the food fortification Regulation (EC) 1925/2006 or the food supplement Directive 2002/46/EC. These regulatory omissions create an oversight because formulators of final foods or supplements can find guidance only in the general horizontal rules of European Union (EU) food law [6]. A recent example of misconceptions associated with amino acid use in nutritional products is the protracted lawsuit between a German pharmaceutical company and the national government on the safety of the essential amino acid histidine [10]. Only for a very narrow category of foods intended for both growing children and adults, the so-called “Foods for Specific Groups”, there is a specific list of amino acids (Regulation (EU) 609/2013). However, the list does not provide any standards of purity.

It is likely due to this absence of specific EU rules that regular food products fortified with amino acids to improve protein quality require a pre-market notification from the EU member state where they are introduced. If amino acids were used in foods for other purposes, the regulatory approach would be similar, and a notification from the member state that is the primary marketing target would be required. In such cases, attention would have to be paid to the final product falling, or not falling, under the novel food category established by the Commission Implementing Regulation (EU) 2017/2470. Comparable to the situation observed in food category, individual member states of the EU implement divergent approaches to how proteinogenic amino acids are regulated in dietary supplements. In most member states, there are no specific rules. Only a few member states (e.g., Spain, the Netherlands, and Denmark [11,12]) have implemented positive lists and maximum daily intakes for amino acids. Switzerland (a non-EU country) has been using a similar approach since 2018 [13]. To the best of our knowledge, only the regulatory framework of Denmark includes purity standards for amino acids [12].

In the United States (US), the 21 Code of Federal Regulations (Section 172.320) defines the addition of amino acids to regular foods to improve protein quality. Other uses of amino acids in regular foods are not precluded but require specific determinations of the “generally recognized as safe” (GRAS) status of each individual amino acid added [14], because the US Food and Drug Administration (FDA) does not encourage GRAS determinations of combinations of nutrients (unpublished data). A GRAS determination, conducted by qualified experts with scientific training and expertise to evaluate the safety of products under their intended condition of use, should consider all relevant safety aspects, including the production methods, dosing, toxicity, and target consumer group(s). As a consequence of that evaluation, maximum daily limits are usually established in a product-specific and target-consumer group-specific manner [15]. A comparable approach to adding (fortifying) amino acids to regular prepackaged foods is also enforced in Brazil, where the fortification with amino acids, which is not intended to improve the protein quality of the final food, leads to a so-called “novel food” classification and necessitates pre-market approval by the National Sanitary Surveillance Agency [16].

Other than the US, EU, and Brazil, most countries do not have specific regulatory tools to deal with the addition (fortification) of free amino acids to regular foods, even for the purpose of improving the protein quality; in such cases, a premarket notification to the responsible regulatory authority would be recommended. However, there are few exceptions; Australia and Japan are the most visible ones. Australia very strictly limits amino acid fortification in a single food category named “formulated supplementary foods” [17], although this rule is currently being revised (since August 2021). On the other hand, Japan classifies most amino acids among “existing food additives” and allows their use without dose restrictions, except for a few exceptions made of specific forms of amino acids with so-called technological functions (e.g., cysteine HCl).

Unlike regular foods, the use of amino acids in dietary supplements in the US is governed by the US Dietary Supplement Health and Education Act of 1994. Under this statute of US federal legislation, supplements are regulated simply by the FDA for good manufacturing practices under 21 Code of Federal Regulations Part 111. Solely the so-called “new dietary ingredients” are rigorously evaluated in terms of safety before being placed on the market. The 21 Code of Federal Regulations (Section 413(d)) defines “new dietary ingredients” as dietary ingredients that were not marketed in the US in a dietary supplement before 15 October 1994. Safety evaluation of “new dietary ingredients” to be used in dietary supplements should be conducted in a comparable manner to the GRAS determination of novel food ingredients. Due to their long history of safe use, proteinogenic amino acids are frequently used as dietary supplements without federal supervision of the dosing, formulation, or purity standards [7].

Similar to the US, some Asian countries (e.g., Japan, Korea, Malaysia, and Singapore) do not supervise amino acid use in dietary supplements, while others (e.g., Indonesia, India, Thailand, and Taiwan) implement positive lists together with maximum daily doses for each of the listed amino acids, e.g., [18]. In Latin America, there was an absence of regulatory supervision concerning dietary supplements until 2018, when Brazilian authorities implemented a positive list of amino acids in dietary supplements with individual maximum limits that were substantially higher than those from some EU member states [11,12,13,19]. For example, the essential amino acid leucine in dietary supplements is subject to a daily limit of 5.6 g in Brazil, 3.0 g in Spain, and 1.3 g in Denmark [11,12,19]. These differences illustrate the differences in the adoption of the precautionary principle, e.g., [20,21], because no adverse effects from supplemental leucine (up to >20 g per person) have been identified in controlled human studies conducted in several age groups [22,23]. Some other Latin American countries, notably Argentina and Uruguay, are adopting a similar risk assessment approach to amino acids as Brazil, hopefully with a view of harmonizing both the risk assessment and regulatory oversights across all Mercosur countries.

To summarize, globally, there are significant disparities in the regulatory approaches to adding amino acids as nutrients to foods or using them in dietary supplements, and there have been minimal efforts to harmonize such approaches on the global level (*Codex Alimentarius*) or even regional levels (e.g., in the EU). The risk management agencies that consider proteinogenic amino acids as a potential source of health risk due to the uncontrolled intake of dietary supplements or foods tend to regulate their use by restricting the maximum limits of intake, even though no cases of overdosing with dietary amino acids were reported in the peer-reviewed literature, e.g., [24]. This approach has three problems: (1) a lack of human safety data combined with conflicting applications of the precautionary principle of the existing data; (2) a lack of purity data for the amino acids that were studied at high-intake doses in humans (in other words, it is unknown whether the observed adverse effects (if any) were caused by the studied amino acid or an impurity); and (3) practical hurdles to enforcing the daily maximum limits of intake. On the other hand, impurities in nutrients have been reported to be causative factors in past outbreaks of diseases, e.g., [4,25], and thus they should be a direct target of regulatory risk management. This is especially true considering the current increase in the global e-commerce of finished products because an absence of purity standards applicable to nutrients in one country, together with a lack of international harmonization, may represent a health risk to the final consumer in another country. Below, we summarize some of the current know-how in analytical chemistry for commercially used amino acids, and attempt to draw some recommendations.

## 3. Analytical Methods to Determine Impurities in Amino Acids

### 3.1. Introduction to Analytical Methods

Since the mid-1950s, commercially used amino acids have been produced mostly by chemical/enzymatic synthesis or fermentation [26]. Therefore, the most observed impurities are those produced through the various reaction pathways during synthesis and/or fermentation, or those that result from inefficient purification steps [27]. Compounds other than those in the amino group, such as fermentation-derived products and compounds created by chemical conversion from amino acids, may be present as impurities. In addition, most of the amino acids and their impurities are hydrophilic and are not retained in the reverse-phase analytical column, so some ingenuity regarding their analytical conditions is required for their separation.

The task of analytically separating impurities is complex, and various analytical methods have been reported (Table 1). The most frequently applied methodology is high-performance liquid chromatography (HPLC). A cation exchange column [28], reverse-phase column [29,30,31,32], hydrophilic interaction liquid chromatography (HILIC)-mode column [33,34], and mix-mode column [35] are used for HPLC separation. In addition, ion-pair mobile phases are applied to detect amino acid impurities with a reverse-phase column [36,37,38,39] or HILIC-mode column [40]. Most amino acids and impurities that lack a chromophore are detected by ultraviolet (UV) detection at a low wavelength near UV 210 nm [3,29,30,33,36] or charged aerosol detection (CAD) [29,37,38,39,40]. For more sensitive detection, pre-column derivatization with fluorescence reagents and fluorescence detection (FL) are used.

In addition to HPLC, there have been some reports of using capillary electrophoresis (CE) [41] and micellar electrokinetic chromatography (MEKC) [42,43,44,45]. CE has a high separation efficiency for charged analytes, and MEKC enables the separation of uncharged analytes using micelle-forming agents, such as sodium dodecyl sulfate. To identify the chemical structure of impurities, mass spectrometry (MS) and nuclear magnetic resonance are most often applied [3,35,38].

### 3.2. High-Performance Liquid Chromatography

**Figure 1:**
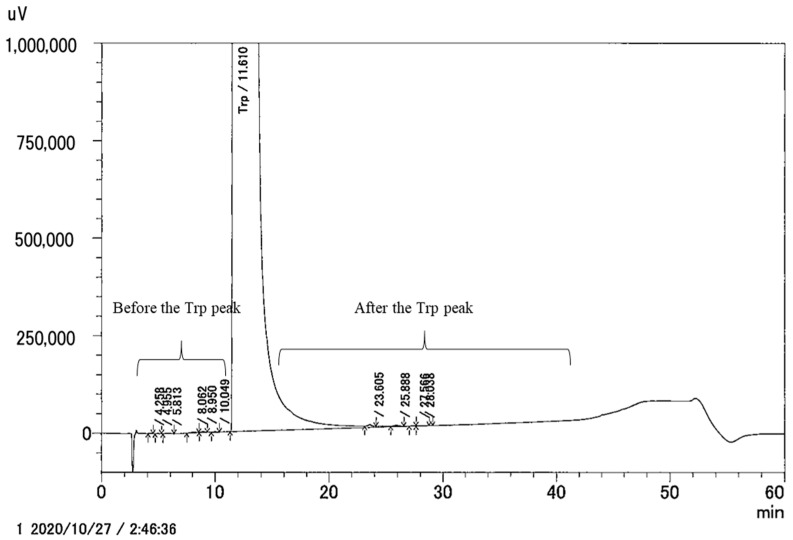
UV (220 nm) chromatograms of Trp dietary supplements. The analysis was performed based on the FCC monograph (12th edition). Reprinted with permission from Ref. [3].

The HPLC methodology coupled with UV, FL, CAD, and MS detection is currently the main method used for the analysis of amino acid impurities. Amino acid analyzers, based on cation-exchange chromatography separation followed by post-column derivatization with ninhydrin and UV detection, are commonly employed. Using the amino acid analyzer, amino group-containing impurities can be detected [28]. Various analytical methods using reverse phase (C18) columns have also been reported. Among others, Pawellek et al. used a polar-embedded C18 column (Acclaim™ Polar Advantage II) coupled with CAD and UV detection for the analysis of impurities in aspartic acid and glycine [29]. Since CAD can detect compounds that lack a chromophore and do not absorb UV, impurities can be comprehensively detected. Kühnreich et al. reported an analytical method using a reverse-phase column with embedded acidic ion-pairing groups (Primesep^®^ 100, SIELC), which they applied to a methionine purity analysis [30]. Recently, Karakawa et al. [3] performed HPLC according to the methodology described in the FCC monograph (12th edition) for the analysis of total tryptophan impurities (Figure 1 and Table 2). In the FCC monograph, the acceptance criteria of total amount of impurities (converted to N-acetyl-Trp) before the Trp peak is 100 ppm and after the Trp peak is 300 ppm. The authors reported substantial differences in the purity of the analyzed dietary Trp supplements [3].

Wahl et al. used a mixed-mode column combining hydrophobic C18 and strong cation exchange retention mechanisms and evaluated cysteine from three different manufacturers [35]. An ion-pair mobile phase and reverse-phase column are often used in combination. Schilling et al. reported an analytical method using a C18 column with an ion-pair reagent (sodium octanesulfonate) and applied it to asparagine analysis [36]. Qiu et al. reported the use of trifluoroacetic acid and heptafluorobutyric acid (HFBA) as ion-pair reagents [37]. Holzgrabe et al. reported that PFHA forms an ion pair with an amino group to enable the retention of amino acids in reverse-phase columns [38,39]. Recently, the HILIC mode, a mode that retains more hydrophilic compounds, has been increasingly used for amino acids and small peptides [33,34,40].

### 3.3. Capillary Electrophoresis

The CE methodology is suitable for the separation of hydrophilic compounds and is also used for the analysis of amino acids and their impurities. A non-labeled method was developed and applied to glutathione impurity analysis [41]. Novatchev et al. reported a MEKC method for the analysis of phenylalanine, serine, tryptophan impurities in samples from several manufacturers using derivatization with 9-fluorenylmethyl chloroformate and UV detection at 254 nm [42]. In addition, the MEKC method was also reported for analyzing the impurity profiles of arginine. In this method, 3-(4-carboxybenzoyl)quinoline-2-carboxaldehyde was used for the labeling of primary amines, and laser-induced fluorescence detection was applied for sensitive detection [43,44,45].

### 3.4. Chiral Analysis

For impurity profiling of amino acids, determination of the enantiomeric purity is another important factor for a review; see [46,49]. Especially, chemically synthesized amino acids contain D-amino acids, and these amino acid have different biological functions in the human body. Recent advances in the sensitive detection of amino acid enantiomers were mostly based on the combination of pre-column derivatization with liquid chromatograph–tandem mass spectrometry (LC/MS/MS) determination [47,48].

### 3.5. Mass Spectrometry Detection

The MS methodology enables the more selective detection of impurities than UV and fluorescent detection, and the LC/MS/MS methodology was successfully applied for the simultaneous detection of several impurities in commercial products [31]. In terms of amino acids, applications of LC/MS/MS have mainly been reported for the determination of the cause(s) of eosinophilia-myalgia syndrome in users of the adulterated tryptophan product in the 1990s [50,51,52,53,54,55]. As a result of analytical work in the 1990s, the possible triggers of the eosinophilia-myalgia syndrome were narrowed down to two impurities: 1,1’-ethylidenebis-L-tryptophan (EBT) and 3-(phenylamino)alanine (PAA). Consequently, Food Chemicals Codex (FCC) monographs have specified since the mid-1990s that both EBT and PAA should not be detected in any tryptophan product intended for human consumption [3].

A recently developed analytical method for the determination of hydrophilic metabolites by LC/MS/MS [32] was used by the current authors to detect impurities in five dietary supplements containing branched-chain amino acids (BCAA) purchased online during April–May 2022 (product information can be obtained from the corresponding author upon request). In Table 3, the chemical names of impurities, estimated from their accurate mass, and peak area are shown. Impurity peak area, in each of the evaluated supplement brands, is expressed as a relative number to highlight substantial differences in purity levels. Although most impurities detected (Table 3) are other amino group-containing compounds which are not toxic per se, their high concentration indicates compromised purification control, and thus points towards a possible risk. Moreover, as mentioned in the introduction, there are no toxicological experimental data on interaction of impurities and specific amino acids, which is another point of concern.

## 4. Discussion and Recommendations

The most frequently applied analytical methodologies, namely HPLC and CE, appear to be able to precisely separate total impurities in proteinogenic amino acids (Table 1), but they cannot comprehensively separate impurities in all amino acids. Thus, it is necessary to select the most suitable method for the amino acid to be analyzed, or to use multiple separation modes and detection methods, which is difficult and costly when analyzing diverse amino acids. This challenge is made more difficult by the most proteinogenic amino acids, except for tryptophan, tyrosine, and histidine, not absorbing UV well. Therefore, methods of derivatizing amino groups with a fluorescent reagent are used, although derivatization detects only compounds containing an amino group, and its overall performance is therefore insufficient. Even if there have been no severe adverse health effects attributed to impurities in amino acids during the last decade [6], it is important to determine impurities as a proactive way to detect abnormalities in quality before adverse health effects occur [3,4,5,6,7,8]. Hence, it is desirable to set a standard analytical method that is internationally recognized, validated, and used. Recently, a group of industrial experts from the “Amino Acid Coalition”, a coalition formed by six major international trade associations, e.g., [56], attempted to review and recommend a set of specifications (monographs): focusing on the FCC and European Pharmacopeia (EP) monographs. The FCC is a compendium of standards for the identity, purity, and quality of food ingredients used in international commerce. In 2006, publication of the FCC was assumed by the United States Pharmacopeia (USP) Convention, a non-governmental standards-setting organization. On the other hand, the EP is a European reference compendium for the quality control of raw materials used in the production of medicines, intermediates of synthesis, and in final medicines.

The “Amino Acid Coalition” found that chemical identification of amino acids was described in more detail in the EP monographs than in the FCC monographs. Some typical contaminants covered by the EP monograph, but not by the FCC monographs, were chlorides, sulfates, ammonium, and iron. However, limits for general parameters, such as loss of drying, sulfated ash, or content (assay), were often identical or only slightly differed between the two monographs. Notably, heavy metals were not listed in the EP monographs, because ingredients used in the pharmaceutical field must comply with the Guideline for Elemental Impurities (ICH Q3D). These experts did not endorse either set of specifications but noted that the organizations that assume the responsibility for establishing and validating specifications (monographs) for food ingredients work with the same intent to ensure the safety and quality of food products. As described in the general principles of the FCC [57], ingredient specifications “*are designed to ensure that food ingredients have the specified identity and a sufficiently high level of quality to be safe under usual conditions of intended use in foods or in food processing*”. Because the objectives are identical, the above-described differences in the specifications should not be interpreted as deficiencies. Instead, the specifications established by internationally recognized organizations should be viewed as equivalent in terms of ensuring the quality and safety of food ingredients.

From a regulatory perspective, there are three takeaways: (1) analyzing amino acid impurities is methodologically a complex issue (Table 1) and there is currently no single comprehensive method to carry this out for groups of amino acids at the same time. (2) This review (Figure 1, Table 2 and Table 3) and previous analyses of commercial products, e.g., [3,33], have found wide variations in the impurity profiles of amino acids and very limited efforts to reduce impurity levels. (3) Amino acid specifications established by internationally recognized organizations, such as the FCC, and EP, provide a comparable level of protection against major breaches of purity.

Because of the complexity of analytical chemistry, managing health risks by adopting national legislation, such as one of the international purity standards (e.g., the FCC), is the most viable current option for managing health risk, e.g., [3,23]. To be even more effective, we recommend that international purity standards (monographs) should describe production methodologies with critical control points, namely purification steps in the case of industrial fermentation. Indeed, most of the commercially used amino acids or vitamins are made by fermentation and purification/crystallization cycles determine the final level of impurities. Therefore, outlining a minimum level of purification/crystallization for each amino acid in an international monograph would go a long way to ensuring the safety of the final ingredient.

Such an approach is advantageous in that it is enforceable because checking purity is subject to analytical confirmation (see Section 3 above), whereas controlling the daily maximum intake is not. For example, a dietary supplement may contain one third of the daily maximum limit for a specific amino acid, but it is impossible to control the number of supplements consumed. One can argue that the precautionary principle [20] is implemented to account for such uncertainty, and that the daily maximum limits for nutrients are therefore not set at their true toxicological maximum limits [7,21]. Although the precautionary principle is a valid approach to managing unknown risks, there are three reasons why it should not be applied to managing health risks of amino acids by establishing restrictive maximum daily limits, as follows. (1) There have been no peer-reviewed reports of health damage caused by an amino acid overdose [6,24], and there are no differences in the reported adverse effects between countries that strictly regulate the maximum daily doses and those that do not. (2) Using rodent toxicological data, obtained with nutrients ingested by humans at more than 1 g/day, is burdened by nutrient-non-specific complications; therefore, it is inadequate as a risk assessment approach, e.g., [58,59]. (3) Limiting the dose of an amino acid to the extent that a dietary supplement is ineffective without a health risk rationale equates to misleading the final consumer, who may end up purchasing a product that is not effective for its advertised function due to low dosing.

Therefore, if the precautionary principle is to be applied, it would be more efficient to apply it to the purity of amino acids. Truly, the findings reported here (Table 2 and Table 3) add to the existing body of peer-reviewed literature, e.g., [3,8,33,42,43] and show that there is a disparity in the purity of major commercial amino acids, such as tryptophan, arginine and branched-chain amino acids, indicating a potential health hazard to the final consumer. The main drawback of regulating purity would be a possible marginal increase of costs of final products due to a “entry barrier” for cheap ingredients, some of them currently derived from human hair or even produced for farm animal use [7].

It has previously been postulated that a proactive and targeted scientific approach is necessary to avoid health risks derived from nutrients, especially during times of escalating costs for science and health care [7,8]. Here, we further recommend that (1) the targeted approach should be re-focused on identifying comprehensive analytical methods that can separate impurities and that (2) the regulatory risk management of nature-identical nutrients should be based on analytical chemistry of the nutrients’ purity, rather than on the unenforceable application of the precautionary principle of maximum daily dosing.

## Figures and Tables

**Table 1:** Summary of analytical methods for detecting amino acid impurities.

Analytical Methodology		Separation Mode	Detection	LOD or LOQ	Amino Acid	Sample Type	Impurities	Ref
HPLC	cation exchange	cation exchange column with post column derivatization with ninhydrine	UV (570 nm, 440 nm)	not described	lysine, methionine, threonine	feed grade amino acids, and premixes	not described	[28]
	C18	a polar embedded C18 column (Acclaim™ Polar Advantage II)	UV (210 nm) and CAD	LOQ 0.02–0.05%	aspartic acid and glycine	analytical grade, synthesis grade	aspartic acid impurity (alanine, asparagine, fumaric acid, glutamic acid, maleic acid, and malic acid), glycine impurity (sarcosin)	[29]
	C18	a reverse-phase analytical column with embedded acidic ion-pairing groups (Primesep^®^ 100)	UV (210 nm)	LOD 0.06–0.30 μg/mL (0.0004–0.002%)	methionine	chemical reagents	l-methionine-sulfoxide and N-acetyl-dl-methionine	[30]
	C18	C18 column	UV (280 nm) and MS (SRM)	LOD 1.3 ng/mL for levodopa impurity B; 5.26 ng/mL for levodopa impurity C; 0.833 ng/mL for methyldopa; 3.31 ng/mL for methylcarbidopa; 1.67 ng/mL for entacapone impurity C; 0.61 ng/mL for entacapone impurity A.	3,4-dihydroxyphenylalanine (Dopa)	film-coated tablets	levodopa impurity B, levodopa impurity, methyldopa, methylcarbidopa, entacapone impurity C, entacapone impurity A.	[31]
	C18	C18 column	UV (220 nm)	not described	tryptophan	nine commercial Trp dietary supplements	1,1′-ethylidenebis-L-tryptophan (EBT), 2-[2,3-dihydroxy-1-(3-indolyl)-propyl-L-tryptophan (dhPIT)	[3]
	PFP	pentafluorophenylpropyl (PFP) column	MS	LOD 1–39 nmol/L	all proteinogenic amino acids	chemical reagents	not described	[32]
	HILIC	Kinetex core-shell 2.6 μm HILIC column	UV (200 nm)	LOQ 1.3 µg/mL	glutathione	dietary supplements	oxidized glutathione	[33]
		Intrada Amino Acid column	MS	not described	17 proteinogenic amino acids	standard solution	glutamic acid was degraded to pyroglutamic acid in 0.1N HCl.	[34]
	mix mode (reversed phase and cationic exchange)	mixed mode column combining hydrophobic C18 and strong cation exchange retention mechanisms	mass spectrometer	LOD 0.03%	carbocysteine	six batches of three different manufacturers	cystine and N,S-dicarboxymethylcysteine	[35]
	ion pair chromatography (IPC)	C18 column with ion pair reagent (sodium octanesulfonate)	UV (210 nm)	LOD 0.025%	asparagine	produced from several manufacturers	Diketoasparagine, aspartic acid	[36]
	ion pair chromatography (IPC)	C18 AQ cloumn with ion pair reagent (trifluoroacetic acid (TFA) and heptafluorobutyric acid (HFBA))	CAD, MS	LOD 0.02%	6 proteinogenic amino acids	injection	9 impurities	[37]
	ion pair chromatography (IPC)	Inertsil ODS 3 column with ion pair reagent (PFHA)	NQAD, CAD, ELSD, MS, NMR	not described	alanine	pharmaceutical grade	aspartic acid, glutamic acid	[38]
	ion pair chromatography (IPC)	Inertsil ODS 3 column with ion pair reagent (PFHA)	CAD	LOD 0.03%	alanine, aspartic acid	samples of pharmaceutical grade aspartic acid and alanine (various manufacturers)	aspartic acid impurity (malic acid and alanine), alanine impurity (aspartic acid, glutamic acid)	[39]
	ion pair chromatography (IPC), and HILIC	Acclaim Polar Advantage II column with ion pair ragent (HFBA and TFA) or Accucore™ 150 Amide HILIC column	CAD	LOD 3 ng on column	leucine, isoleucine, and valine (BCAA)	not described	alanine, cysteine, methionine, leucine, isoleucine, valine, phenylalanine	[40]
CE	CE	fused-silica capillaries	UV (200 nm)	LOD 0.01%	glutathione	three batches produced from one manufacturer	oxidized glutathione, glutamylcystein, cysteinylglycine and cysteine	[41]
	MEKC	fused-silica capillaries with pre-column derivatization using FMOC	UV (254 nm)	LOD 0.1%	phenylalanine, serine, and tryptophan samples	produced from several manufacturers	phenylalanine impurity (isoleucine and leucine), serine and tryptophan impurity (not identified)	[42]
	MEKC	fused-silica capillaries with pre-column derivatization using FMOC or CBQCA	laser induced fluorescence (LIF) detection (em. 488 nm, ex. 520 nm)	LOD >0.05%	histidine, isoleucine, phenylalanine	produced from several manufacturers	histidine impurity (not identified), isoleucine impurity (glycine, valine, leucine, alanine), phenylalanine impurity (tyrosine)	[43]
	MEKC	fused-silica capillaries with derivatization using fluorescamine (FLA)	UV (254 nm)	LOD 0.1 µmol/L levels	tryptophan	medical nutrition	5-methyl-L-tryptophan, 1-methyl-L-tryptophan, 5-hydroxy-L-tryptophan	[44]
	MEKC	fused-silica capillaries with pre-column derivatization using CBQCA	laser induced fluorescence (LIF) detection (em. 488 nm, ex. 520 nm)	LOD 0.1% w/w	arginine	produced by fermentation (various manufacturers)	amino sugars, low molecular peptides and amino acids	[45]
Chiral separation	HPLC	Daicel Crownpak CR(+) with post-column derivatization with OPA	FL (ex 340nm, em450nm) and UV (200nm),	LOD 0.001% (10 ppm)	alanine, phenylalanine, aspartic acid, threonine, leucine	chemical reagents	D-amino acids	[46]
		Phenyl column and pre-column derivatization with (R)-BiAC	MS (SRM)	LOD Attomole to subfemtomole order on column	19 proteinogenic amino acids	chemical reagents	D-amino acids	[47,48]
	CE	direct approach: chiral selectors indirect approach: chiral reagents	UV and FL	not described	all proteinogenic amino acids	chemical reagents	D-amino acids	[49]

**Table 2:** Total impurities in the tested tryptophan (Trp) products. Reprinted with permission from Ref. [3].

	Total Impurities (ppm)	
No.	Before the Trp Peak	After the Trp Peak
1	182.5	199.1
2	97.6	123
3	517.7	846.7
4	73.5	7.3
5	18.5	182.5
6	13.9	202.7
7	80.5	359.7
8	931.4	161.6
9	88.4	317.1

**Table 3:** The individual impurities and their peak area detected by LC/MS in dietary supplements containing branched-chain amino acid (BCAA). Samples were dissolved in water and prepared at final concentrations equal to 1 mg/mL.

			Peak Area Are of Impurity				
Detected Imputities	Calculated MW	Accurate Mass	Supplement 1	Supplement 2	Supplement 3	Supplement 4	Supplement 5
Phenylalanine	165.07884	166.08612	4,476,043,398	1,147,667,894	6,528,709,427	1,516,625,946	26,789,958
a-Aminobutyric Acid	103.06319	104.07046	20,553,802	1,602,835,019	2,188,738,731	1,270,456,055	10,852,005
Tyrosine	181.07383	182.08111	139,765,018	141,281,087	963,463,526	348,594,566	3,594,945
Methionine	149.05099	150.05827	1,013,198,876	488,098,943	92,087,336	398,158,949	21,341,637
Lysine	146.10541	147.11269	333,230,158	377,853,088	273,390,570	206,379,227	8,260,686
Homocystine	268.05467	269.06195	390,439	88,562,248	230,961,786	786,141	554,987
Glutamic Acid	147.05306	148.06034	71,668,419	81,819,720	166,587,109	25,110,649	28,056,457
Homolanthionine	236.08277	237.09005	15,113,251	81,165,087	59,474,555	160,681,729	2,154,190
Xanthine	152.03339	153.04067	466,191	71,971,417	246,248,479	452,885	421,383
Serine	105.0425	106.04978	3,885,912	6,168,018	4,651,923	27,327,075	2,153,298
Glutamine	146.06905	147.07633	1,830,905	25,159,071	2,354,309	2,363,000	6,770,331
Guanine	151.04938	152.05666	594,482	2,573,421	45,615,044	42,861,831	549,337

## Data Availability

Information on supplements analyzed can be obtained from the corresponding author upon request.
